# The Prevalence of Sleep Disturbances and Anxiety Among Croatian University Students: Possible Association with Lifestyle Factors and COVID-19

**DOI:** 10.3390/jcm15062157

**Published:** 2026-03-12

**Authors:** Tina Zavidić, Ema Dejhalla, Ana Lesac Brizić, Tatjana Čulina, Branislava Popović

**Affiliations:** 1 Department of Family Medicine, Faculty of Medicine, University of Rijeka, 51000 Rijeka, Croatia; ana.lesac@outlook.hr (A.L.B.); tatjana.culina@zzjzpgz.hr (T.Č.); branislava.popovic@medri.uniri.hr (B.P.); 2 Medical Centre for Occupational Health Rijeka, Family Medicine Office, 51000 Rijeka, Croatia; emadejhalla@gmail.com

**Keywords:** insomnia, anxiety, students, sleep disorders, COVID-19

## Abstract

**Background/Objectives**: University students are particularly vulnerable to psychological distress and sleep disturbances, which may impair academic performance and overall well-being. Lifestyle changes, increased academic demands, and the COVID-19 pandemic may have further exacerbated these problems. This study aimed to assess the prevalence of insomnia and anxiety among students at the University of Rijeka and to examine their associations with COVID-19 infection, vaccination status, lifestyle habits, and sociodemographic characteristics. **Methods**: A cross-sectional online survey was conducted between October 2024 and April 2025 among 594 students from 14 faculties of the University of Rijeka. Data were collected using a self-administered questionnaire including sociodemographic variables, the Insomnia Severity Index (ISI), the Generalized Anxiety Disorder-7 (GAD-7), lifestyle behaviors, and COVID-19-related factors. Statistical significance was set at *p* < 0.05. **Results**: Insomnia was reported by 50.7% of students (36.9% mild, 12.1% moderate, 1.7% severe), while 49.6% reported elevated anxiety levels. Female sex, smoking, low physical activity, poor subjective sleep quality, and frequent nighttime awakenings were significantly associated with higher ISI and GAD-7 scores (all *p* < 0.05). COVID-19 infection and vaccination status showed weaker associations with insomnia compared to psychosocial and lifestyle factors. Students living in shared rental accommodation reported higher insomnia severity. In multivariable regression analysis, anxiety severity, sleep fragmentation (frequent nocturnal awakenings), and poor perceived sleep quality emerged as the strongest independent predictors of insomnia severity. **Conclusions**: Insomnia and anxiety are highly prevalent among University of Rijeka students and are primarily associated with psychosocial and lifestyle factors rather than COVID-19-related variables. University-based interventions focusing on stress reduction, sleep hygiene, and early mental health support are warranted, particularly for high-risk groups.

## 1. Introduction

University life represents a critical developmental period characterized by increasing academic demands, psychosocial stressors, and greater autonomy in lifestyle choices [1,2]. During this transition, health-related behaviors—particularly sleep hygiene—are often neglected, despite their fundamental role in mental health, cognitive functioning, and academic performance [3]. University students are therefore recognized as a population at elevated risk for both sleep disturbances and psychological distress [4].

Global evidence consistently demonstrates a high mental health burden among university students, with prevalence rates of anxiety and depressive symptoms approaching 50% in some regions [5,6]. Similarly, poor sleep quality and insufficient sleep duration are highly prevalent, affecting approximately half of the student population worldwide [7]. These conditions frequently co-occur and exhibit a bidirectional relationship: sleep disturbances can exacerbate anxiety and mood symptoms, while psychological distress can further impair sleep initiation and maintenance [8,9]. Although this interplay has been widely acknowledged, its determinants and relative contributions remain incompletely understood, particularly in non-clinical student populations [10].

Sleep plays a central role in emotional regulation, cognitive performance, and stress resilience [11]. Chronic sleep deprivation and sleep fragmentation have been associated with impaired attention, reduced academic achievement, emotional dysregulation, and increased vulnerability to anxiety disorders [12,13]. Longitudinal studies further suggest that sleep disturbances emerging during adolescence and early adulthood may predict the later development of psychiatric conditions, highlighting the importance of early identification and prevention [14].

The COVID-19 pandemic introduced additional stressors that may have amplified these existing vulnerabilities. Public health measures such as lockdowns, social distancing, and transitions to online education disrupted daily routines and circadian rhythms, contributing to a marked global increase in sleep disturbances—often referred to as “COVIDsomnia” [15,16]. Meta-analyses estimate that up to 40% of the general population experienced sleep problems during the pandemic, with young adults being particularly affected [17]. Individuals infected with SARS-CoV-2 have reported even higher rates of sleep disturbances, both during acute illness and in the post-infection period, raising concerns about persistent neuropsychiatric and circadian effects [18]. Emerging evidence also indicates that some individuals experience prolonged post-COVID symptoms, including fatigue, sleep disturbances, cognitive difficulties, and anxiety months after the initial infection, even among younger and otherwise healthy populations [19,20,21,22]. These long-term sequelae have raised concerns regarding potential lasting impacts on mental health and daily functioning in young adults, including university students [19].

In parallel, large-scale COVID-19 vaccination campaigns raised questions regarding the interaction between sleep, immune function, and vaccine response. Growing evidence suggests that insufficient or disrupted sleep may impair immunological responses to vaccination, resulting in reduced antibody production [23,24,25]. However, findings remain heterogeneous, and data addressing these associations in young, generally healthy populations—such as university students—are limited [26].

Previous studies have examined associations between sleep disturbances, psychological distress, and pandemic-related factors among university students [27,28,29]. However, findings vary across populations and geographical regions, and systematic reviews indicate substantial cross-cultural differences in prevalence estimates [30]. In addition, relatively few studies have examined these factors together with lifestyle behaviors and sociodemographic characteristics within the same analytical framework in the later stages of the pandemic period [30,31,32].

Therefore, the present study aimed to determine the prevalence of insomnia and anxiety among students at the University of Rijeka and to examine their associations with COVID-19-related factors, lifestyle habits, and sociodemographic characteristics. We further examined whether insomnia and anxiety were more strongly associated with psychosocial and lifestyle factors than with COVID-19 infection or vaccination status. By addressing these factors within a Croatian university population, this study provides additional context-specific evidence on student sleep and mental health in the post-pandemic period and may help inform targeted, university-based interventions aimed at improving sleep, mental health, and overall student well-being.

## 2. Materials and Methods

### 2.1. Aim, Study Design, and Setting

The aim of this study was to determine the prevalence of insomnia among students at the University of Rijeka and to examine its associations with anxiety symptoms, COVID-19 infection, COVID-19 vaccination status, lifestyle habits, and sociodemographic characteristics. A cross-sectional study was conducted between October 2024 and April 2025 across 14 faculties of the University of Rijeka, Croatia.

### 2.2. Participants and Recruitment

A total of 594 students participated in the study. Participants were recruited using a convenience sampling approach through official university mailing lists, student organization communication channels, and university-affiliated social media platforms. Of the total sample, 65.2% were female and 34.8% were male. Most participants enrolled at the university in 2019 (20.9%), 2020 (17.5%), 2021 (16.7%), or 2023 (15.1%). Inclusion criteria were: (1) current enrollment in undergraduate or postgraduate programs at the University of Rijeka, (2) age ≥ 18 years, and (3) provision of informed consent. The exclusion criteria were as follows: age < 18 years; diagnosed insomnia, anxiety or depressive disorder; severe psychiatric diagnoses or recent traumatic events. Responses were excluded if the questionnaire was incomplete or if participants did not meet the inclusion criteria.

### 2.3. Data Collection Procedure

Data were collected anonymously using an online, self-administered questionnaire created with Google Forms (Google LLC, Mountain View, CA, USA). The introductory page provided detailed information about the study aims, ensured confidentiality and anonymity, and informed participants of their right to withdraw at any time without consequences. Completion time was approximately 10 min. No personally identifiable information was collected.

### 2.4. Survey Instruments

The questionnaire consisted of four sections:

1. Sociodemographic characteristics, including age, sex, study program, year of study, and living arrangement.
2. Insomnia symptoms, assessed using the Insomnia Severity Index (ISI), a validated 7-item self-report scale measuring the severity of insomnia symptoms over the previous two weeks. Each item is scored on a 5-point Likert scale, yielding a total score ranging from 0 to 28,with higher total scores indicating greater insomnia severity. Total scores are interpreted as follows: 0–7 (no clinically significant insomnia), 8–14 (subthreshold insomnia), 15–21 (moderate insomnia), and 22–28 (severe insomnia) [33].
3. Anxiety symptoms, assessed using the Generalized Anxiety Disorder-7 (GAD-7) questionnaire, a widely used 7-item instrument evaluating anxiety symptoms experienced during the previous two weeks. Each item is rated on a 4-point Likert scale (0–3), resulting in a total score ranging from 0 to 21, with higher scores indicating greater anxiety severity. Total scores are interpreted as follows: 0–4 (minimal anxiety), 5–9 (mild anxiety), 10–14 (moderate anxiety), and 15–21 (severe anxiety) [34].
4. Comorbidities and COVID-19-related items, including self-reported history of COVID-19 infection, COVID-19 vaccination status, perceived pandemic-related stress, self-rated sleep quality, frequency of nighttime awakenings, lifestyle habits (e.g., smoking, physical activity), and self-perceived changes in sleep and mental health during the pandemic period.

### 2.5. Statistical Analysis

Statistical analyses were performed using IBM SPSS Statistics 26.0 (IBM Corp., Armonk, NY, USA). Descriptive statistics were used to summarize participant characteristics and questionnaire scores. Categorical variables were analyzed using the chi-square test. When expected cell frequencies were less than five, Fisher’s exact test was applied to ensure the validity of statistical inference. Where necessary, categories with very small subgroup sizes were combined to meet test assumptions. Continuous variables were analyzed using non-parametric tests, specifically the Mann–Whitney U test or the Kruskal–Wallis test, as appropriate. Spearman correlation coefficients were calculated to assess associations between continuous variables. Multivariable regression analyses were conducted using appropriate non-parametric approaches to identify independent predictors of insomnia severity (ISI score). Statistical significance was set at *p* < 0.05.

### 2.6. Ethical Considerations

The study protocol was reviewed and approved by the Ethics Committee of the University of Rijeka (Approval No. 641-01/23-01/80, 2170-1-42-08-3-23-1; approved on 20 April 2023). The study was conducted in accordance with the Declaration of Helsinki (2013 revision). All participants provided informed consent prior to participation.

## 3. Results

**Figure 1:**
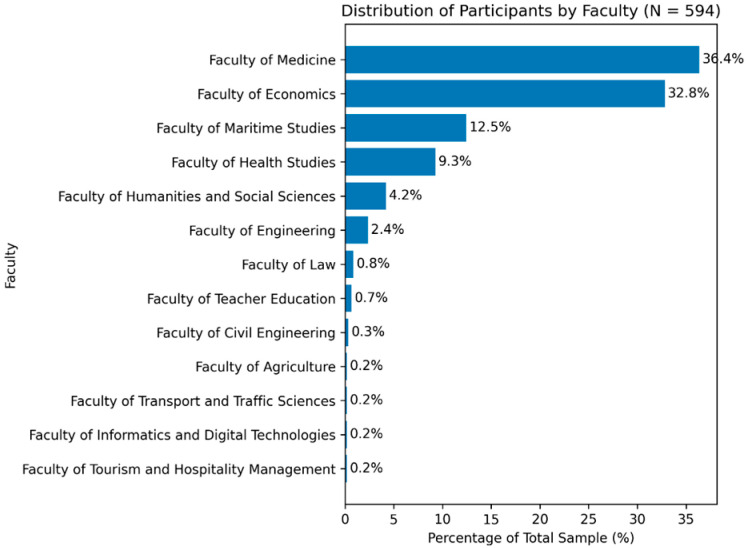
Participation of individual faculties in the total study population.

A total of 594 students participated in the study, of whom 65.2% were female and 34.8% male. This sex distribution reflects the overall demographic structure of the University of Rijeka student population. Participants were predominantly young adults (mean age 27 ± 4.7) with a mean body mass index (BMI) of 23.65 ± 3.70 kg/m^2^, corresponding to the normal weight range. Students from all university faculties were represented, most frequently from the Faculty of Medicine and the Faculty of Economics, followed by the Faculty of Maritime Studies and the Faculty of Health Studies (Figure 1).

### 3.1. Insomnia Severity Index Questionnaire

The participants’ responses on the Insomnia Severity Index (ISI) are summarized in Table 1. Regarding difficulty falling asleep, 32.8% reported no problems, while 35.7% reported frequent problems. Problems with staying asleep were reported as none by 42.9% of participants and moderate or more severe by 29.5%. Early awakening was the least common sleep disturbance, with 52.4% reporting no problems, though 28.4% indicated frequent or very severe issues (Table 1).

Regarding satisfaction with sleep patterns, 39.2% of respondents were satisfied, while 29.9% reported being dissatisfied. Sleep problems did not impact daily functioning in 26.8% of participants. but impacts a lot for 19.4%. When asked about how noticeable their sleep problems were to others, 40.1% reported that others did not notice them at all, whereas 4.8% felt their sleep difficulties were frequently observed. Finally, nearly half of the participants (47.1%) reported no concern about their sleep problems, while 8.5% were considerably worried (Table 1).

### 3.2. Generalized Anxiety Disorder-7 (GAD-7)

Table 2 presents the prevalence of anxiety symptoms measured by the GAD-7 scale over the past two weeks. Items are scored from 0 to 3 (0 = not at all; 3 = nearly every day). Higher scores indicate greater anxiety severity. The most commonly reported problems were excessive worry (68.9%), difficulty relaxing (64%), and feeling nervous, anxious, or on edge (63.5%). More than half of the respondents reported an inability to control worrying (57.9%) and sudden feelings of anger or irritability (51.8%). Additionally, a third of respondents felt so restless that it was difficult to sit still and also reported feeling that something awful might happen.

As presented in Table 3A,B, nearly half of the participants (49.3%) reported no clinically significant insomnia according to the ISI, while 36.9% reported mild insomnia, 12.1% moderate insomnia, and 1.7% severe insomnia. In contrast, the majority of participants reported no clinically significant anxiety based on GAD-7 scores (50.3%). Mild anxiety was observed in 32.8% of students, moderate anxiety in 9.4%, and 7.4% participants reported severe anxiety. These findings indicate that approximately half of the participants reported experiencing sleep and anxiety difficulties, with 50.7% indicating some form of insomnia and 49.6% reporting some form of anxiety.

Normality of the ISI and GAD-7 total scores was assessed using the Kolmogorov–Smirnov and Shapiro–Wilk tests. Both tests indicated that the distributions of ISI (K-S = 0.101, *p* < 0.001; S-W = 0.956, *p* < 0.001) and GAD-7 (K-S = 0.157, *p* < 0.001; S-W = 0.866, *p* < 0.001) scores significantly deviated from a normal distribution. Consequently, non-parametric statistical methods were deemed appropriate for subsequent analyses.

Spearman’s rank-order correlation was used to examine the relationship between insomnia severity (ISI total score) and anxiety severity (GAD-7 total score). Results indicated a moderate, positive, and statistically significant correlation between ISI and GAD-7 scores (r = 0.546, *p* < 0.001), suggesting that higher levels of insomnia were associated with higher levels of anxiety in this student sample.

Analysis of GAP-7 categories revealed significant associations between anxiety and multiple demographic, lifestyle, and clinical factors (Table 4). Female participants and current smokers were more likely to report mild or moderate anxiety. Lack of physical activity was also associated with higher anxiety severity. Living arrangements played a role: participants living in shared housing reported more severe anxiety, whereas those living with parents or in student dormitories or alone exhibited lower prevalence.

Subjective sleep quality and anxiety were strongly linked to anxiety severity, with participants perceiving poor sleep or experiencing anxiety showing higher GAP-7 scores. Specific sleep disturbances—including difficulty falling asleep, frequent night awakenings, sleepwalking, restless legs, bruxism, and night sweating—were significantly more common among participants with mild or moderate insomnia.

Patterns of sleep disturbances, including frequency, duration, and onset, were associated with anxiety severity also. Participants experiencing frequent disturbances, longer duration of problems (>6 month), use of sleep medication, shorter sleep duration (<4–6 h), increased nocturnal awakenings (>5 times), and shorter time to fall back asleep after awakenings (>30 min) were more likely to report higher anxiety severity. Sleep problems at the beginning of studies or associated with stressful events, such as family or personal issues, were also associated with higher GAD-7 scores, including among participants who had received COVID-19 vaccination.

Finally, participants who did not communicate about their sleep problems with others exhibited greater anxiety severity. If they communicate, it was most often with their friends or parents.

COVID-19 infection history differs significantly by anxiety severity (*p* = 0.036), with single infections most common in normal and mild anxiety groups, two infections more frequent in moderate anxiety, and three infections more common in severe anxiety, revealing distinct group-specific patterns.

A higher proportion of female reported mild, moderate, or severe insomnia (Table 4). Almost all participants with moderate or severe insomnia perceived their sleep quality as poor. Anxiety was more prevalent among participants with mild, moderate, and severe insomnia. Most sleep disturbances were statistically significant (*p* < 0.05): difficulty falling asleep, frequent awakenings and early morning awakenings were notable in all categories of insomnia; breathing pauses were more common in moderate insomnia as well as restless legs; snoring and night sweating were significantly associated with more severe insomnia; early morning awakenings were notable in all categories of insomnia.

The duration and frequency of insomnia were also related to severity. Participants with more severe insomnia experienced longer-lasting sleep problems, and although the use of sleep medications was rare, it was more common among those with moderate and severe insomnia. Total sleep duration decreased with increasing insomnia severity, and nighttime awakenings were more frequent in moderate and severe insomnia.

Regarding triggers, the onset of university studies, stressful life events (family or personal), and after COVID-19 vaccination were significant contributors to insomnia.

Finally, the majority of participants with severe insomnia had not discussed their sleep problems with anyone. Among those who did seek support, conversations were most often held with friends, physicians, or parents.

## 4. Discussion

This study demonstrates a high prevalence of both insomnia and anxiety symptoms among students at the University of Rijeka, supporting the growing body of international evidence indicating that university students represent a particularly vulnerable population with respect to mental health and sleep disturbances. Nearly half of the participants reported clinically relevant anxiety symptoms, while more than 40% experienced insomnia. These findings are consistent with recent meta-analyses reporting elevated rates of anxiety and poor sleep quality among undergraduate students worldwide and confirm our initial hypothesis that sleep and anxiety problems remain highly prevalent in the post-pandemic period [35].

Although the COVID-19 pandemic has been identified as a major contributor to psychological distress and sleep disruption, the present findings suggest that, in this sample, insomnia and anxiety were more strongly associated with psychosocial and lifestyle-related factors than with COVID-19 infection or vaccination status. It is possible that pandemic-related effects were transient and attenuated over time or that unmeasured factors such as preexisting mental health conditions, social support, or economic stressors contributed to the observed symptomatology. Thus, causality cannot be inferred from these cross-sectional data, and the associations may be influenced by unmeasured confounders.

Sex differences emerged as a consistent and robust finding, with female students reporting significantly higher anxiety and insomnia severity. This pattern has been widely documented in previous studies and has been attributed to biological differences in stress responsivity, hormonal influences, emotion regulation, and sociocultural expectations. These results highlight the importance of sex-sensitive screening and prevention strategies in university health programs [36,37,38], but further research is needed to clarify causal mechanisms.

Lifestyle behaviors also showed notable associations with mental health. Smoking showed a trend toward a higher prevalence of insomnia, although this did not reach statistical significance. Also, smoking was strongly linked to elevated anxiety, consistent with studies from Canadian student cohorts reporting that tobacco use is often intertwined with psychological distress [39]. Although alcohol and marijuana use were not significantly associated with anxiety or insomnia in this sample, earlier studies indicate that substance use may both reflect and exacerbate stress in young adults, highlighting the need to consider contextual and temporal factors when interpreting these findings. A study of medical students in Poland found that variables such as smoking cigarettes, consuming energy drinks several times a month, or daily stress had a negative impact on the quality of sleep of medical students [40]. Among 400 young adults daily-level estimates showed increased alcohol use was associated with poorer perceived sleep health, while stronger effects from marijuana were associated with better perceived sleep health [41]. 

Physical activity demonstrated a beneficial trend, supporting evidence that regular exercise enhances emotional regulation, reduces physiological arousal, and improves sleep continuity [42]. Insufficient physical activity (<150 min/week) was more common among students with more severe anxiety, whereas those with normal findings were more likely to engage in ≥150 min of weekly activity. Physical activity, while not statistically significant, suggested a shift in students with insomnia toward lower activity levels, consistent with the well-established role of physical activity in sleep regulation. Nevertheless, self-reported activity levels may be subject to recall bias, and causality cannot be inferred.

Subjective sleep characteristics—including poor perceived sleep quality, difficulties initiating and maintaining sleep, non-restorative sleep, and frequent nighttime awakenings—were among the strongest correlates of both anxiety and insomnia severity. These findings reinforce the well-established bidirectional relationship between sleep and psychological well-being and are consistent with previous studies showing that students with higher anxiety are substantially more likely to report poor sleep quality and daytime impairment [29,43,44]. Still, it remains unclear whether poor sleep is a cause, consequence, or part of a cyclical interaction with anxiety. Longitudinal studies are required to disentangle these pathways.

Environmental and psychosocial factors also played a significant role. Sleep problems frequently emerged during the transition to university, and students living in shared rental accommodation reported greater insomnia severity, suggesting that changes in routine, academic demands, and living environments may contribute to sleep disruption [45].

Additionally, symptoms suggestive of sleep-disordered breathing, such as snoring and nocturnal breathing pauses, were associated with greater insomnia severity, highlighting the need to consider undiagnosed sleep disorders even in young, non-clinical populations [46,47]. These findings highlight the multifactorial nature of sleep disturbances but also emphasize that residual confounding by unmeasured variables, such as stress-coping styles, social support, or comorbid psychiatric conditions, cannot be ruled out.

Most somatic health conditions were not associated with insomnia, with the exception of certain categories such as mental health disorders (depression, anxiety, other psychiatric conditions), urinary problems and sensory system disorders (vision or hearing-related problems). This supports the view that sleep disturbances among students are more strongly tied to psychosocial than to physical health factors. This is consistent with previous research showing that academic stress, lifestyle transitions, and environmental factors, such as living arrangements and social pressures, are key contributors to poor sleep quality among students [45,48]. Our findings suggest that sex-specific patterns in insomnia and anxiety may reflect broader interactions between sleep, lifestyle, and health outcomes. These patterns are consistent with previously reported associations between sleep and somatic health indicators in young adults [48]. However, underreporting or undiagnosed somatic conditions could have influenced these associations.

Patterns of help-seeking varied across insomnia severity. Students with moderate-to-severe symptoms were more likely to discuss sleep problems with others and to use sleep medications or natural sleep aids, sometimes without medical guidance. This raises concerns regarding unsupervised pharmacological use and suggests that students may lack access to appropriate sleep and mental health support. Overall, these findings highlight the need for integrated university-based interventions targeting stress reduction, sleep hygiene, and early mental-health screening.

### 4.1. Limitations

Several limitations should be acknowledged. The cross-sectional design precludes causal inference regarding the directionality of associations between sleep, anxiety, and lifestyle factors. All data were self-reported and therefore subject to recall and reporting bias, particularly with respect to sleep characteristics and COVID-19 history. Online recruitment may have introduced self-selection bias, potentially overrepresenting students with sleep or mental health concerns. Finally, although validated screening instruments were used, clinical diagnostic interviews were not conducted.

The study also did not specifically assess persistent post-COVID sequelae (e.g., long COVID symptoms). Although COVID-19 infection history was recorded, it was not possible to distinguish between students who fully recovered and those experiencing ongoing post-infectious symptoms. Future research should compare students without prior infection, those with resolved infection, and those with persistent post-COVID manifestations to better clarify the long-term impact of SARS-CoV-2 on sleep and mental health outcomes.

### 4.2. Implications and Future Directions

Despite these limitations, this study provides valuable insight into the multifactorial nature of sleep and anxiety problems among university students in Croatia. The findings underscore the need for integrated, university-based interventions focusing on sleep hygiene education, stress management, promotion of physical activity, and early mental health screening.

Future longitudinal studies are warranted to clarify causal pathways and to evaluate the effectiveness of targeted prevention and intervention strategies aimed at improving both sleep and psychological well-being in student populations.

## 5. Conclusions

Sleep disturbances are highly prevalent among students at the University of Rijeka and are closely associated with anxiety symptoms, lifestyle behaviors, and key sociodemographic factors. Sleep fragmentation and poor subjective sleep quality emerged as central contributors to insomnia severity and impaired daily functioning, underscoring the clinical relevance of subjective sleep complaints in student populations. Observed differences by sex and academic context highlight the need for tailored, population-specific approaches. Overall, these findings emphasize the importance of implementing targeted university-based interventions focused on stress reduction, sleep hygiene education, and early mental health support to improve student well-being and inform institutional health policies.

## Figures and Tables

**Table 1:** Distribution of Insomnia Severity Index (ISI) item responses (n= 594).

ISI Item	None n (%)	Mild n (%)	Moderate n (%)	Severe/Very Severe n (%)	Mean ± SD *
Difficulty falling asleep	195 (32.8)	187 (31.5)	127 (21.4)	85 (14.3)	1.20 ± 1.10
Difficulty staying asleep	255 (42.9)	164 (27.6)	110 (18.5)	65 (10.9)	0.99 ± 1.07
Early morning awakening	311 (52.4)	114 (19.2)	78 (13.1)	91 (15.3)	0.96 ± 1.23
Sleep satisfaction	38 (6.4) †	195 (32.8)	183 (30.8)	178 (30.0)	1.88 ± 1.00

* Mean total item score (range 0–4; higher scores indicate greater symptom severity). † Very satisfied category shown separately where applicable.

**Table 2:** Distribution of responses to the Generalized Anxiety Disorder-7 (GAD-7) items (N = 594).

GAD-7 Item	Not at All n (%)	Several Days n (%)	More Than 7 Days n (%)	Nearly Every Day n (%)	Mean ± SD
Feeling nervous, anxious, or on edge	217 (36.5)	284 (47.8)	19 (3.2)	74 (12.5)	0.92 ± 0.94
Not being able to stop or control worrying	250 (42.1)	261 (43.9)	26 (4.4)	57 (9.6)	0.81 ± 0.90
Worrying too much about different things	185 (31.1)	297 (50.0)	38 (6.4)	74 (12.5)	1.00 ± 0.94
Trouble relaxing	214 (36.0)	275 (46.3)	34 (5.7)	71 (12.0)	0.94 ± 0.95
Being so restless that it is hard to sit still	402 (67.7)	139 (23.4)	17 (2.9)	36 (6.1)	0.47 ± 0.82
Becoming easily annoyed or irritable	286 (48.1)	240 (40.4)	27 (4.5)	41 (6.9)	0.70 ± 0.85
Feeling afraid, as if something awful might happen	381 (64.1)	163 (27.4)	16 (2.7)	34 (5.7)	0.50 ± 0.81

**Table 3:** Severity categories of insomnia (ISI) and anxiety (GAD-7) (N = 594). Severity classification based on validated cut-off scores.

**(A) Insomnia Severity Index (ISI)**		
**ISI Category**	**Score Range**	**n (%)**
No clinically significant insomnia	0–7	293 (49.3)
Mild insomnia	8–14	219 (36.9)
Moderate insomnia	15–21	72 (12.1)
Severe insomnia	22–28	10 (1.7)
**(B) Generalized Anxiety Disorder-7 (GAD-7)**		
**GAD-7 Category**	**Score Range**	**n (%)**
Minimal anxiety	0–4	299 (50.3)
Mild anxiety	5–9	195 (32.8)
Moderate anxiety	10–14	56 (9.4)
Severe anxiety	15–21	44 (7.4)

**Table 4:** Bivariate associations of selected variables with anxiety (GAD-7) and insomnia (ISI) severity.

Variable	Anxiety Severity (GAD-7), *p* *	Insomnia Severity (ISI), *p* *
Sociodemographic factors		
Sex	<0.001	0.004
Living arrangement	0.001	ns
Lifestyle factors		
Smoking	0.003	ns
Physical activity	<0.001	ns
Subjective perception		
Poor sleep quality	<0.001	<0.001
Self-perceived anxiety	<0.001	<0.001
Specific sleep disturbances		
Difficulty falling asleep	<0.001	<0.001
Frequent night awakenings	<0.001	<0.001
Early morning awakening	0.018	<0.001
Restless legs	<0.001	0.030
Night sweats	<0.001	<0.001
Snoring	ns	<0.001
Breathing pauses	ns	0.009
Sleep characteristics		
Sleep duration	<0.001	<0.001
Nocturnal awakenings frequency	<0.001	<0.001
Time to fall back asleep	<0.001	<0.001
Onset and triggers		
Beginning of university studies	<0.001	<0.001
Beginning of pandemic	ns	0.011
Post-vaccination onset	0.049	0.011
Stressful life events	<0.001	<0.001
Help-seeking behaviour		
Did not discuss sleep problems	<0.001	<0.001
Talked to parents	0.001	<0.001
Talked to friends	<0.001	<0.001
Talked to physician	0.005	<0.001
Medical conditions		
Psychiatric comorbidity	<0.001	<0.001
No recorded disease	<0.001	0.001
COVID-19 variables		
COVID-19 infection frequency	0.036	ns
Vaccination status	ns	0.041

* Chi-square test or Fisher’s exact test, as appropriate, ns = not statistically significant (*p* ≥ 0.05).

## Data Availability

The raw data supporting the conclusions of this article will be made available by the authors on request.

## Abbreviations

The following abbreviations are used in this manuscript:

GAD-7	Generalized Anxiety Disorder-7
ISI	Insomnia Severity Index
COVID-19	Coronavirus disease 2019
BMI	Body Mass Index
